# FUT2‐Mediated Fucosylation of OLFM4 Promotes Colon Cancer Cell Differentiation

**DOI:** 10.1155/cjgh/7502916

**Published:** 2026-06-30

**Authors:** Caihan Duan, Keyi Zhang, Lingzhi Hou, Jiawei Chen, Jun Liu, Huiying Shi, Chaoqun Han

**Affiliations:** ^1^ Division of Gastroenterology, Union Hospital, Tongji Medical College, Huazhong University of Science and Technology, Wuhan, Hubei, China, hust.edu.cn

**Keywords:** colon cancer cell, differentiation, fucosylation, fucosyltransferase 2 (FUT2), olfactomedin-4 (OLFM4)

## Abstract

**Background:**

Fucosyltransferase 2 (FUT2) deficiency exacerbates inflammation, a known risk factor for colon cancer. However, the precise role and underlying mechanism of FUT2 in regulating colon cancer cell differentiation remain unclear. Although olfactomedin‐4 (OLFM4) has been known to have a tumor‐suppressive effect, its functional interaction with FUT2 in colon cancer remains unexplored.

**Methods:**

FUT2 expression levels were assessed using TCGA and cBioPortal databases and in different colon cancer cell lines. ALP assays were performed to evaluate the effect of FUT2 on cancer cell differentiation. Transwell invasion assays, scratch assays, and tumor sphere formation assays were used to investigate the functional effects of FUT2 on colon cancer cells. N‐glycosylation proteomics and UEA‐I chromatography were used to identify the regulatory effect of FUT2 on OLFM4 fucosylation.

**Results:**

FUT2 expression was associated with the differentiation degree of colon cancer cell lines. Based on their endogenous FUT2 expression, we overexpressed FUT2 in SW480 cells (low baseline) and knocked it down in HT29 cells (high baseline). Overexpressing FUT2 promoted the differentiation of SW480 cells and inhibited their migration, invasion, EMT, and stemness. Conversely, knockdown of FUT2 in HT29 cells reduced its degree of differentiation and increased its malignancy. N‐glycosylation proteomics analysis and UEA‐I chromatography indicated OLFM4 as a downstream target of FUT2. FUT2‐mediated fucosylation of OLFM4 is positively correlated with the degree of cancer cell differentiation. Knockdown of OLFM4 attenuated differentiation in FUT2‐overexpressing SW480 cells, while overexpression of OLFM4 produced opposite effects.

**Conclusion:**

FUT2 promotes colon cancer cell differentiation by mediating OLFM4 fucosylation, suggesting that FUT2 may serve as a therapeutic target for colon cancer.

## 1. Introduction

Colon cancer is a leading cause of cancer‐related mortality worldwide, driven by its rapid progression, frequent late‐stage diagnosis, and broad metastatic potential to sites like the liver, lungs, ovaries, and other gastrointestinal organs [1, 2].

Deficiencies in fucosylation, a process mediated in intestinal epithelial cells by enzymes such as fucosyltransferase 2 (FUT2), have been linked to colitis and adenocarcinoma in mice [3–5]. FUT2 protects the intestine via fucosylation [6], and its deficiency exacerbates colon inflammation [7], a known risk factor for colon cancer [8]. However, the specific role of FUT2 in colorectal cancer (CRC) remains unclear.

Olfactomedin‐4 (OLFM4), a glycoprotein highly expressed in gastrointestinal tissues and bone marrow [9], has emerged as a key regulator in cancer biology [10, 11]. Its expression is associated with cell differentiation, and it exerts complex, context‐dependent roles in tumorigenesis. In CRC, OLFM4 is generally regarded as a marker of differentiation, and its loss is frequently linked to malignant progression [12–14]. For instance, *in vitro* functional studies demonstrate that downregulation of OLFM4 enhances proliferative and migratory capacities of colon cancer cells [15, 16], and OLFM4‐positive colon cancer patients exhibit significantly better prognoses than their OLFM4‐negative counterparts [17]. Mechanistically, OLFM4 has been shown to suppress epithelial‐to‐mesenchymal transition (EMT) and maintain an epithelial phenotype, thereby inhibiting metastasis [18, 19]. Despite these advances, the upstream regulatory mechanisms, particularly post‐translational modifications such as fucosylation, that control OLFM4’s function in colon cancer are poorly understood. The potential regulation of OLFM4 by FUT2‐mediated fucosylation and its functional consequences in colon cancer cell differentiation represent a significant knowledge gap.

This study investigates the association between FUT2 and colon cancer cell differentiation, with a specific focus on its regulation through OLFM4 fucosylation. Our findings may offer novel insights for colon cancer treatment and research.

## 2. Materials and Methods

### 2.1. Bioinformatic Analysis

Data on FUT2 expression levels in normal tissues and colon cancer tissues were collected and analyzed from the TCGA database (https://tcga-data.nci.nih.gov/tcga/), which includes gene sequencing data derived from surgically resected tissue samples. Gene expression profiling interactive analysis (GEPIA) was used to create box plots. Gene sequencing results and corresponding clinical characteristics of colon cancer patients based on primary colon tumor tissues were collected and analyzed from the cBioPortal database (https://www.cbioportal.org/). The relationship between clinical stage and FUT2 expression level was analyzed accordingly.

### 2.2. Cell Lines and Cell Culture

Human colon cancer cell lines LoVo (RRID: CVCL_0399), SW480 (RRID: CVCL_0546), HCT116 (RRID: CVCL_0291), and HT29 (RRID: CVCL_A8EZ) were purchased from Wuhan Procell Life Science & Technology Co., Ltd., and cultured with DMEM (Thermo Fisher, Waltham, Massachusetts, USA) supplemented with 10% fetal bovine serum (Thermo Fisher) and 100 U/mL penicillin and streptomycin (Thermo Fisher), and the human colon cancer cell line Caco‐2 (RRID: CVCL_0025) was purchased from the American Type Culture Collection (ATCC) and cultured with RPMI1640 medium (Thermo Fisher). These five cell lines were chosen as they represent a spectrum from poorly to well‐differentiated phenotypes (LoVo, SW480, HCT116, HT29, and Caco‐2), as previous studies have reported [20–22]. Cells were maintained in a 5% CO_2_ incubator at 37°C. The cells were routinely examined to exclude mycoplasma contamination.

### 2.3. Colon Cancer Animal Model

*In vivo* colon cancer model was established in mice by intraperitoneal injection of azoxymethane (AOM) and alternate feeding of dextran sodium sulfate (DSS) as follows: On Day 0, AOM was intraperitoneally injected at 10 mg/kg body weight followed by regular drinking water feeding for 1 week; on Day 7, mice were provided a continuous supply of 2.5% DSS solution in their drinking water for 7 days; on Day 14, mice were again given standard drinking water for 2 weeks. The cycle of DSS administration and regular drinking water was repeated for another 2 cycles. After completing modeling, mice were anesthetized by intraperitoneal injection of pentobarbital and sacrificed, and blood and tissue samples were collected for the following experiments.

### 2.4. Construction of Recombinant Lentiviral Vectors and Lentiviral Transfection

The lentiviral vector system and the empty vectors were purchased from the GeneChem Corporation (Shanghai, China). FUT2 and OLFM4 overexpression constructs were generated using the GV492 vector, with the empty vector GV492‐NC as a control. For knockdown experiments, short‐hairpin RNAs targeting FUT2 and OLFM4 were cloned into the GV493 vector, with GV248‐shNC serving as a negative control. SW480 and HT29 cells (5 × 10^5^) were each transfected with the specific virus (GV492 to SW480 cells, GV493 to HT29 cells) at a multiplicity of infection of 20 in the presence of polybrene (5 µg/mL). After 12 h, the supernatant was discarded and replaced by DMEM complete medium containing 10% FBS and 1% penicillin–streptomycin. Expression of FUT2 and OLFM4 in transfected cells was later detected by Western blot (WB).

### 2.5. RNA Extraction and Real‐Time Quantitative PCR (qRT‐PCR)

RNA was extracted from colon cancer cells using TRIzol reagent (Invitrogen) according to the manufacturer’s protocol. Reverse transcription (cDNA) was synthesized from 1 µg of total RNA with Prime Script RT Master Mix (Takara Biotechnology). qRT‐PCR was performed using 1 µL of first‐strand cDNA with the LightCycler 480 SYBR I Master Mix (Roche) at a final volume of 10 µL. All samples were run in triplicate and underwent 45 amplification cycles on a Roche LightCycler 480 system (Roche). The 2−^ΔCT^ method was used to calculate relative gene expression normalized to GAPDH (glyceraldehyde‐3‐phosphate dehydrogenase). Primers used in this study are listed in Table 1.

**TABLE 1:** Primer sequence used for RT‐PCR.

Gene	Upstream primer sequence	Downstream primer sequence
FUT2	GTGGTGTTTGCTGGCGATGG	AAAGATTTTGAGGAAAGGGGAGTCG
OLFM4	AAGGACTGTATTGGGTGGCG	CACAGCAATCGTGTTGGTGG
GAPDH	ACCCACTCCTCCACCTTTGA	AAAGTGGTCGTTGAGGGCAA

### 2.6. WB

Proteins were extracted from colon cancer cells using RIPA Lysis Buffer (Beyotime) supplemented with phenylmethyl sulfonyl fluoride, protease inhibitors, and phosphatase inhibitors, and incubated at 4°C for 20 min. The total protein concentration was determined using a Pierce BCA Protein Assay Kit (Thermo Fisher), and denatured protein samples of appropriate quality were subjected to sodium dodecyl sulfate–polyacrylamide gel electrophoresis and then transferred to polyvinylidene fluoride membranes. Then, membranes were later blocked with 10% skimmed milk and incubated overnight at 4°C with primary specific antibodies against FUT2 (catalog number: A5721, anti‐rabbit), OLFM4 (catalog number: A15387, anti‐rabbit), E‐cadherin (catalog number: A20798, anti‐rabbit), N‐cadherin (catalog number: A0433, anti‐rabbit), vimentin (catalog number: A2584, anti‐rabbit), Snail (catalog number: A5243, anti‐rabbit), β‐tubulin (catalog number: A12289, anti‐rabbit), and GAPDH (catalog number: A19056, anti‐rabbit), and all primary antibodies were purchased from Aibotec Biotechnology Co., Ltd, Wuhan, China. After washing with TBST for three times, 10 min each time, membranes were incubated with horseradish peroxidase (HRP)–conjugated goat anti‐rabbit secondary antibodies (GeneTex, Irvine, California, USA; catalog number: 511203, goat–anti‐rabbit) at a dilution of 1:2000 for 1 h at room temperature. Protein bands were visualized by the FluorChem Imaging System (ProteinSimple) using the commercial Pierce Fast Western Blot Kit and ECL Substrate (Thermo Fisher).

### 2.7. Immunofluorescence (IF) Staining

For IF staining, cell crawling slides were hydrated and treated for antigen retrieval with citrate buffer (pH 6.0), and the slides were fixed in 4% paraformaldehyde for 30 min and then washed with PBS for three times. For Ulex europaeus agglutinin‐I (UEA‐I) staining, sections were incubated with rhodamine UEA‐I for 1 h at 37°C. The nuclei were stained with DAPI (Beyotime) for 8 min at room temperature. Images were acquired using a confocal microscope (Nikon AX R MP).

### 2.8. Scratch Assay

Under aseptic conditions, cells were seeded in six‐well plates at 37°C in a 5% CO2 atmosphere and cultured until cells grew to a density of about 70%∼80% at the bottom of the wells. Straight lines were drawn on the cell layer using a 10‐µL pipette tip. The treated six‐well plates were observed under a microscope after 0, 12, 24, and 48 h, respectively, and photos of the changes of the scratches were taken for further analysis.

### 2.9. Transwell Invasion Assay

Cell invasion assays were performed in a Transwell chamber (Corning). 1 × 10^5^ cells were seeded into the upper chamber containing FBS‐free medium, and the lower chamber contained complete cell culture medium. The upper chambers were precoated with 15 µg/mL Matrigel (Corning). After incubation at 37°C in an atmosphere of 5% CO_2_ for 24 h, the noninvading cells in the upper chamber were removed with cotton swabs. The cells that penetrated the membrane filters were fixed in 4% paraformaldehyde and stained with crystal violet. The number of invaded cells was quantified by counting the number of cells from 10 random fields at × 100 magnification.

### 2.10. Tumor Sphere Formation Assay

Cells (4 × 10^4^) were seeded in six‐well ultra‐low attachment plates per well (Corning) in sphere formation medium: serum‐free DMEM/F‐12 (Invitrogen) supplemented with bFGF (10 ng/mL, Invitrogen), B‐27 (50X) (Invitrogen), human EGF (20 ng/mL, Invitrogen), and IGF (20 ng/mL, Cell Signaling). Cells were subsequently cultured at 37°C in an atmosphere containing 5% CO_2_ to form tumor spheres. After 10–14 days, the images of cells were captured by inverted microscopy at a magnification of × 100.

### 2.11. Alkaline Phosphatase (ALP) Assay

ALP assay was performed using an ALP detection kit (Beyotime Biotechnology) according to the manufacturer’s protocol. Cultured cells were washed once with PBS and lysed with ice‐cold cell lysis buffer. The cells were centrifuged at 4°C for 3–5 min at 12,000 rpm, and the supernatant was collected for the subsequent assay. The excitation wavelength was set to 360 nm and the emission wavelength to 450 nm for fluorescence detection. The fluorescence intensity was compared with a 4‐methylumbelliferone (4‐MU) standard curve to determine ALP activity.

### 2.12. N‐Linked Glycosylation Modification Quantitative Proteomics

N‐linked glycosylation modification quantitative proteomics analysis of the colon of wild‐type mice and intestinal epithelial cell‐specific FUT2‐knockout mice that were used in this study was reported in our previous study [22]. Samples were fractionated by high‐performance liquid chromatography. Tryptic peptide solution was loaded onto the column. Glycopeptides were eluted with 0.1% trifluoroacetic acid, 50 mM ammonium bicarbonate, and 50% acetonitrile (ACN) 2 times, and the samples were dried. Then, the samples were redissolved in 50 mM ammonium bicarbonate solution and digested at 37°C overnight with 2 µL of N‐glycosidase F (PNGase F). Finally, deglycopeptides were desalted by C18 Zip Tips following the manufacturer’s instructions, and the samples were dried for liquid chromatography–tandem mass spectrometry analysis.

### 2.13. UEA‐I Chromatography

UEA‐I chromatography was used to examine the change of protein α1,2‐fucosylation modification. UEA‐I is a lectin that specifically binds to terminal α1,2‐fucosylated glycans, regardless of whether they are attached to N‐linked or O‐linked glycoproteins. The proteins with α1,2‐fucosylation modification were enriched by agarose‐conjugated UEA‐I and then detected by WB. Cells were lysed with ice‐cold cell lysis buffer for WB and immunoprecipitation (Beyotime Biotechnology) to extract the total protein. The protein concentration was confirmed using the BCA method (Servicebio). Cell lysate (500 µg) was mixed with 50 µL of agarose‐bound UEA‐I (Vector Laboratories) and incubated overnight at 4°C with rotation for α1,2‐fucosylated proteins enrichment. Then, the samples were washed 3 times with lysis buffer and centrifuged at 1000 rpm for 2 min at 4°C. UEA‐I‐enriched proteins were extracted by 100°C heating for 10 min with 5 × SDS–polyacrylamide gel electrophoresis loading buffer and centrifuging at 1000 rpm for 2 min.

### 2.14. Statistical Analysis

The numerical results of the data in this study are expressed as mean ± standard deviation, and for statistical differences, one‐way ANOVA or independent *t*‐tests were used to calculate the differences in the data between multiple groups or two groups, respectively. Statistical analysis of data was performed using SPSS Statistics 26 software, and graphs were plotted using ImageJ and GraphPad Prism 8.0 software. *p* < 0.05 was considered a significant difference (^∗^ *p* < 0.05, ^∗∗^ *p* < 0.01, ^∗∗∗^ *p* < 0.001).

## 3. Results

### 3.1. FUT2 Expression Is Associated With Different Degrees of Differentiation in Colon Cancer Cells

**FIGURE 1:**
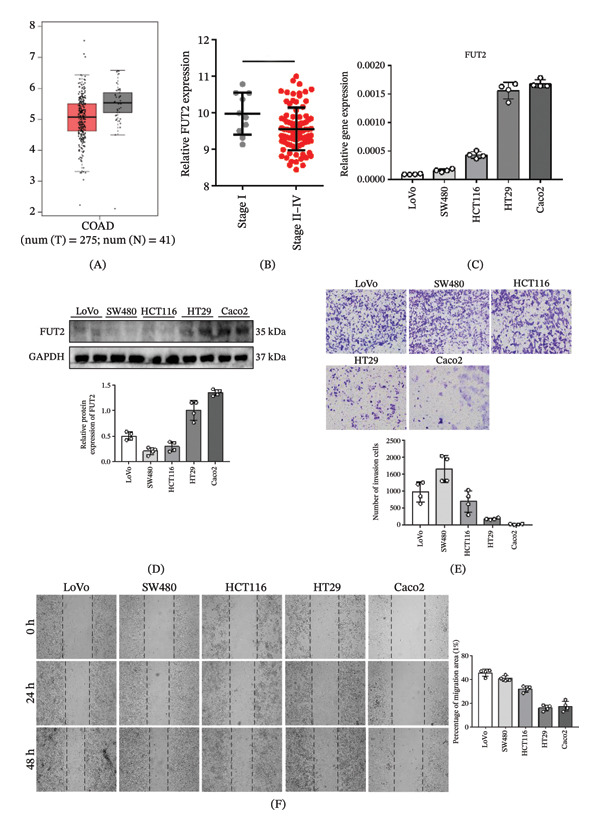
FUT2 expression associates with different degrees of differentiation in colon cancer cells. (A) Bioinformatics analysis of FUT2 expression in normal tissue and colon cancer tissue. FUT2 has a trend of lower expression in colon cancer tissue than in normal tissue, though the result was not statistically significant. (B) Analysis of clinical and sequencing information of colon cancer patients. FUT2 expression level was significantly higher in early‐stage than in advanced‐stage colon cancer tissues. (C) FUT2 RNA expression detected in different colon cancer cell lines by RT‐PCR. The RNA expression level of FUT2 was positively correlated with the degree of differentiation of colon cancer cells. (D) FUT2 protein expression in different colon cancer cell lines detected by Western blot. The protein expression level of FUT2 was positively correlated with the degree of differentiation of colon cancer cells. Transwell invasion assay (E) and scratch assay (F) were performed to evaluate the invasion and migration ability in different cancer cell lines. Among the 5 colon cancer cell lines, migration and invasion of FUT2 high‐expressing HT29 and Caco‐2 cells were relatively inhibited. In contrast, FUT2 low‐expressing LoVo, SW480, and HCT116 cells exhibited more aggressive migrative and invasive potential.

To explore the relationship between FUT2 and colon cancer, we analyzed FUT2 expression in colon tissue samples and normal tissues using data from the TCGA database. While FUT2 expression trended lower in colon cancer tissues compared to normal tissues, this difference lacked statistical significance (Figure 1A). Analysis of clinical and sequencing data (cBioPortal) of colon cancer tissue specimens at different stages revealed significantly higher FUT2 expression in early‐stage versus advanced‐stage colon cancer tissues (*p* < 0.05) (Figure 1B).

We next assessed FUT2 expression across five colon cancer cell lines (LoVo, SW480, HCT116, HT29, Caco‐2) representing a gradient of increasing differentiation (poorly to well‐differentiated). Both RT‐PCR (Figure 1C) and WB (Figure 1D) demonstrated a positive correlation between FUT2 expression levels and the degree of cellular differentiation.

Given established links between colon cancer cell invasion/migration and differentiation status [23, 24], we employed Transwell invasion and scratch assays. Correspondingly, FUT2‐high HT29 and Caco‐2 cells exhibited relatively inhibited migration and invasion, whereas FUT2‐low LoVo, SW480, and HCT116 cells displayed more aggressive potential (Figure 1E,F). Collectively, these results indicate that FUT2 expression is associated with the differentiation status of colon cancer cells.

### 3.2. FUT2 Inhibits Colon Cancer Cell Invasion and Migration

To further confirm FUT2’s role in inhibiting colon cancer cell invasion and migration, we selected low FUT2‐expressing SW480 and high FUT2‐expressing HT29 cells. The selection of SW480 and HT29 cell lines for functional assays was based on their differential expression of FUT2 and their distinct differentiation statuses. These cell lines allowed us to bidirectionally manipulate FUT2 expression and assess functional consequences on cell biological behaviors, optimizing the observable phenotypic range as previous studies have reported [22, 25, 26].

**FIGURE 2:**
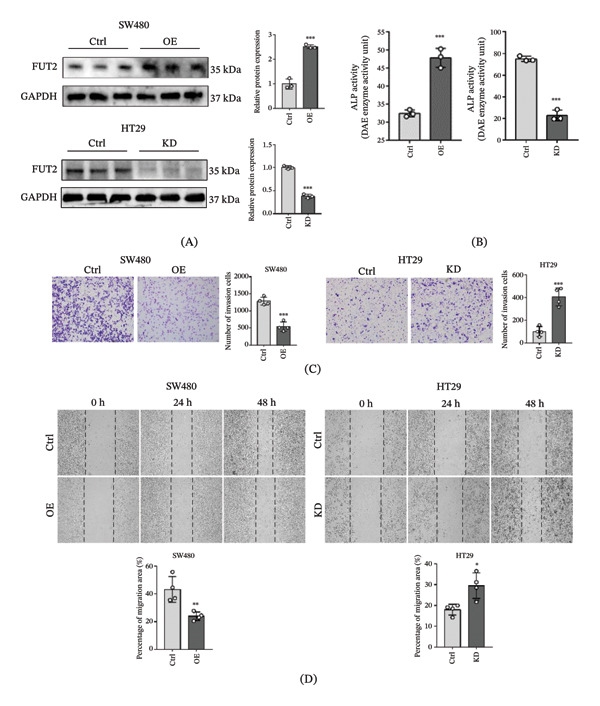
Altered FUT2 expression level affects the invasion and migration of colon cancer cells. (A) Western blot was used to verify the lentiviral transfection effects in SW480 and HT29 cells. Lentiviral transfection overexpressed FUT2 in SW480 cells and knocked down FUT2 in HT29 cells. The transfection was successful. (B) ALP assay suggested FUT2 promoted cancer cell differentiation and inhibited cancer cell stemness. ALP activity was increased in FUT2‐overexpressed cancer cells and inhibited in FUT2‐knockdown cancer cells. Transwell invasion assay (C) and scratch assay (D) were performed and found that the migration and invasion ability of colon cancer cells enhanced in FUT2‐knockdown HT29 cells and decreased in FUT2‐overexpressed SW480 cells.

Lentiviral transfection was used to overexpress FUT2 in SW480 cells and knock down FUT2 in HT29 cells, with transfection efficiency confirmed by WB (Figure 2A). ALP assay, an indicator of differentiation [27], revealed increased ALP activity in FUT2‐overexpressing SW480 cells but decreased activity in FUT2‐knockdown HT29 cells (Figure 2B), suggesting FUT2 promotes differentiation and inhibits stemness. Transwell invasion assays demonstrated inhibited migration and invasion in FUT2‐overexpressing SW480 cells, while FUT2‐knockdown HT29 cells exhibited enhanced invasive potential (Figure 2C). Similarly, scratch assays showed inhibited wound healing in FUT2‐overexpressing SW480 cells and increased healing in FUT2‐knockdown HT29 cells (Figure 2D).

Collectively, these results demonstrate that FUT2 overexpression inhibits invasion and migration, and promotes differentiation in colon cancer cells, while FUT2 knockdown enhances invasion and migration and suppresses differentiation.

### 3.3. FUT2 Regulates Colon Cancer Cell Stemness and EMT

**FIGURE 3:**
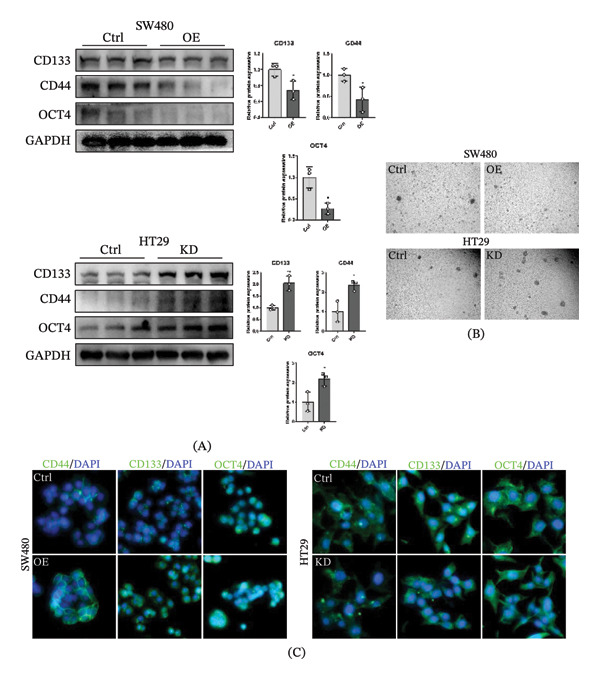
FUT2 regulates colon cancer stemness. (A) Western blot was used to detect stemness biomarkers CD44, CD133, and OCT4 levels in colon cancer cells, and they were downregulated in FUT2‐overexpressed SW480 cells and upregulated in FUT2‐knockdown HT29 cells, respectively. (B) Sphere formation assay was performed and FUT2 overexpression in SW480 cells inhibited sphere formation ability, while FUT2 knockdown enhanced sphere formation ability in HT29 cells. (C) Immunofluorescence staining of stemness markers CD44, CD133, and OCT4. FUT2 overexpression reduced CD44, CD133, and OCT4 fluorescence in SW480 cells, and FUT2 knockdown improved CD44, CD133, and OCT4 fluorescence in HT29 cells.

Stemness markers (CD44, CD133, OCT4) are negatively correlated with colon cancer cell differentiation status [28–30]. To determine whether FUT2 modulates stemness, we assessed CD44, CD133, and OCT4 expression via WB in FUT2‐overexpressing SW480 and FUT2‐knockdown HT29 cells. FUT2 overexpression downregulated, while FUT2 knockdown upregulated, all three stemness markers (Figure 3A). Sphere formation assays confirmed that FUT2 overexpression reduced, and knockdown enhanced sphere formation capacity (Figure 3B). IF staining further demonstrated weakened CD44/CD133/OCT4 signal in FUT2‐overexpressing SW480 cells and intensified signal in FUT2‐knockdown HT29 cells (Figure 3C), collectively indicating FUT2 inhibits colon cancer cell stemness.

**FIGURE 4:**
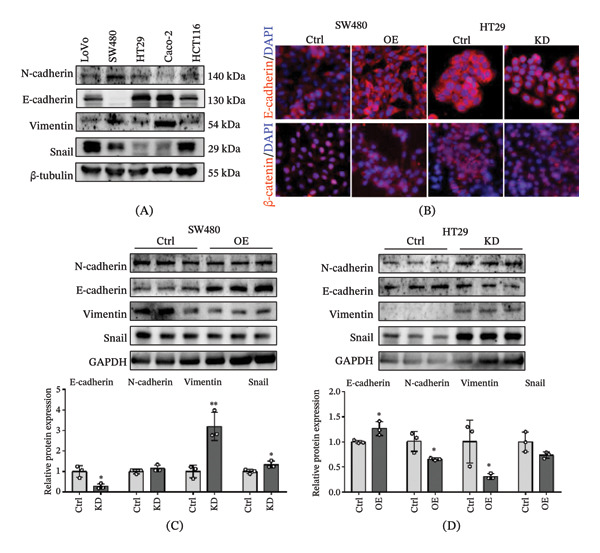
FUT2 inhibits the EMT process of colon cancer cells. (A) The levels of EMT markers (N‐cadherin, E‐cadherin, vimentin, and transcription factor Snail) in cancer cell lines of different differentiation extents were detected by Western blot. N‐cadherin level was found to be negatively correlated with the differentiation extent of colon cancer cells. (B) Immunofluorescence of EMT markers. E‐cadherin was upregulated in FUT2‐overexpressing SW480 cells, while downregulated in FUT2‐knockdown HT29 cells. Conversely, β‐catenin was downregulated in FUT2‐overexpressing SW480 cells, while upregulated in FUT2‐knockdown HT29 cells. The level of EMT‐relevant molecules was detected by Western blot in FUT2‐overexpressed SW480 cells (C) and FUT2‐knockdown HT29 cells (D), respectively. In FUT2‐overexpressing SW480 cells, E‐cadherin expression levels increased (*p* < 0.05), while vimentin and N‐cadherin expression levels decreased (*p* < 0.05). Conversely, in FUT2‐knockdown HT29 cells, E‐cadherin expression levels decreased (*p* < 0.05), whereas vimentin and transcription factor Snail expression levels increased (*p* < 0.05).

EMT also negatively correlates with differentiation status [30]. To assess FUT2’s impact on EMT, we first analyzed EMT markers (including N‐cadherin, E‐cadherin, vimentin, and Snail) across cell lines of varying differentiation. N‐cadherin decreased with increasing differentiation (Figure 4A). IF staining in manipulated cells showed FUT2 overexpression upregulated E‐cadherin and downregulated β‐catenin in SW480 cells, while FUT2 knockdown had the opposite effect in HT29 cells (Figure 4B). WB analysis (Figure 4C,D) corroborated these findings FUT2 overexpression in SW480 cells increased E‐cadherin and decreased vimentin/N‐cadherin; conversely, FUT2 knockdown in HT29 cells decreased E‐cadherin and increased vimentin/Snail. Thus, FUT2 inhibits EMT in colon cancer cells.

### 3.4. FUT2 Upregulates the Fucosylation of OLFM4

**FIGURE 5:**
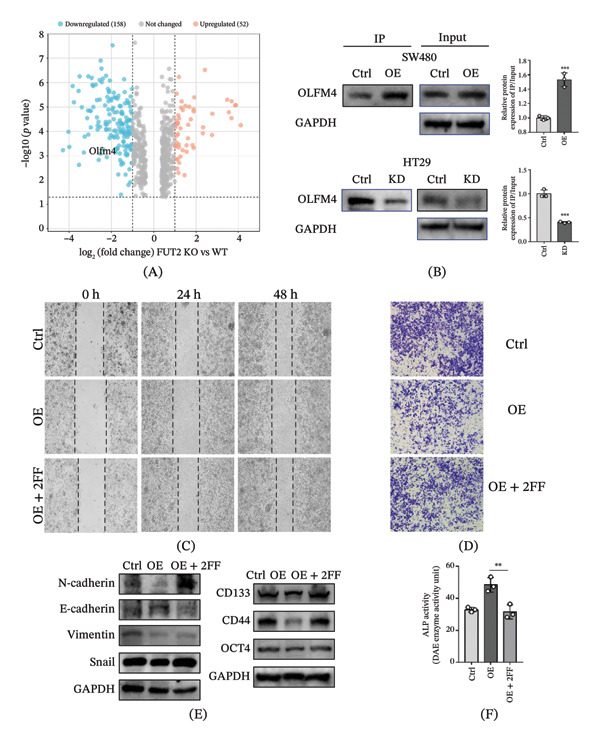
FUT2 upregulates the fucosylation of OLFM4. (A) N‐glycoproteomics assay was performed and revealed a significant decrease in the N‐glycosylation level of OLFM4 in colon cancer tissues. (B) UEA‐I lectin pull‐down and Western blot were performed and confirmed that the fucosylation modification level of OLFM4 was elevated in the FUT2‐overexpressing SW480 cells (*p* < 0.001), while the expression level of fucosylated OLFM4 was decreased in FUT2‐knockdown HT29 cells (*p* < 0.001). Scratch assay (C) and transwell invasion assay (D) were performed in SW480 cells. FUT2 overexpression inhibited the invasive and migratory ability of colon cancer cells, while the application of 2‐fluorofucose (2FF, a type of fucosylation inhibitor) can reverse such an effect and restore the invasive and metastatic ability. (E) Markers of stemness and the EMT process were also detected by Western blot, respectively. E‐cadherin expression was initially increased in FUT2‐overexpressing colon cancer cells and decreased when 2FF was added. In contrast, N‐cadherin and CD44 expression were initially decreased in FUT2‐overexpressing colon cancer cells and increased when 2FF was applied. (F) ALP assay was performed and found that FUT2 overexpression promoted cell differentiation of SW480 colon cancer cells, while application of 2FF can reverse such effect and inhibit cell differentiation.

To identify FUT2 targets influencing colon cancer cell differentiation, we performed N‐glycoproteomics on colon cancer tissues from WT and intestinal epithelial cell‐specific FUT2 knockout (FUT2^ΔIEC^) mice. This revealed significantly reduced N‐glycosylation of OLFM4 in the FUT2^ΔIEC^ group (Figure 5A).

Subsequently, UEA‐I lectin enrichment of fucosylated proteins in FUT2‐overexpressing SW480 and FUT2‐knockdown HT29 cells, followed by WB for enriched and total proteins (Figure 5B), confirmed elevated OLFM4 fucosylation in FUT2‐overexpressing cells and reduced fucosylated OLFM4 in FUT2‐knockdown cells. This verifies FUT2 enhances OLFM4 fucosylation.

To assess the functional role of FUT2‐mediated fucosylation, we inhibited fucosylation using 2‐fluorofucose (2FF). FUT2 overexpression inhibited SW480 cell invasion and migration (Transwell/scratch assays), and 2FF treatment reversed this inhibition (Figure 5C,D). WB analysis showed FUT2 overexpression initially increased E‐cadherin and decreased N‐cadherin and CD44; 2FF reversed these effects (Figure 5E). Similarly, ALP assays indicated FUT2 overexpression promoted SW480 differentiation, which was suppressed by 2FF (Figure 5F).

### 3.5. OLFM4 Is a Downstream Target of FUT2 in Regulating the Differentiation of Colon Cancer Cells

OLFM4 expression positively correlated with colon cancer cell differentiation extent across cell lines, as confirmed by PCR (Figure 6A) and WB (Figure 6B).

FIGURE 6OLFM4 is a downstream target of FUT2 in regulating the differentiation of colon cancer cells. qRT‐PCR (A) and Western blot (B) were performed, and OLFM4 expression was found to be positively correlated with the differentiation extent of cancer colon cells. Plasmid transfection was used to downregulate OLFM4 expression level in FUT2‐overexpressing SW480 cells and to upregulate OLFM4 expression in FUT2‐knockdown HT29 cells in order to investigate whether FUT2 inhibits the invasion and metastasis of colon cancer cells through the OLFM4‐FUT2‐stemness axis, and the transfection was successful as detected by Western blot (C). (D) ALP assay showed that OLFM4 overexpression in FUT2‐knockdown HT29 cells increased differentiation, whereas OLFM4 knockdown in FUT2‐overexpressing SW480 cells attenuated it. Scratch assay (E) and transwell invasion assay (F) were performed in the transfected colon cancer cells, suggesting that upregulation of the expression of OLFM4 inhibits the invasive and metastatic ability of colon cancer cells. (G) EMT markers (N‐cadherin, E‐cadherin, vimentin, and Snail) were detected by Western blot and found that the EMT process was strengthened in FUT2‐overexpressing SW480 cells with inhibited OLFM4 expression. (H) Immunofluorescence staining was performed to investigate the effect of OLFM4 on the process of EMT. Fluorescence of E‐cadherin was weakened in FUT2‐overexpressing SW480 cells with inhibited OLFM4 expression and was increased in FUT2‐knockdown HT29 cells with overexpressing OLFM4, suggesting that OLFM4 could inhibit EMT. (I) Expression of stemness markers (CD44, CD133, OCT4) and tumorigenesis markers was detected by Western blot and found that cancer cell stemness was also strengthened in FUT2‐overexpressing SW480 cells with inhibited OLFM4 expression.

**Figure:**
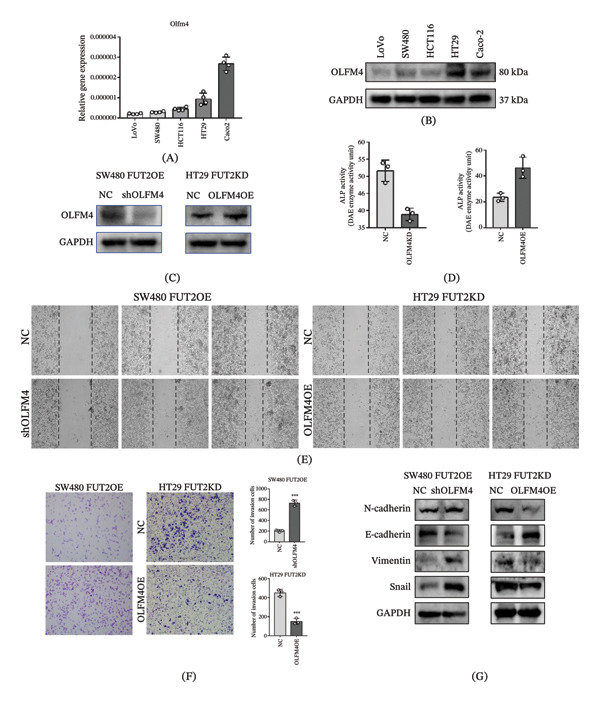

**Figure:**
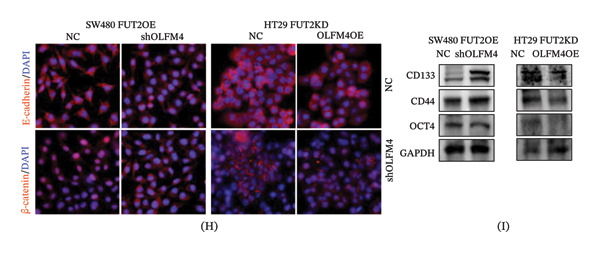

To determine whether FUT2 regulates differentiation via OLFM4, we transfected FUT2‐overexpressing SW480 cells to knock down OLFM4 and FUT2‐knockdown HT29 cells to overexpress OLFM4. Successful transfection was confirmed by WB (Figure 6C).

ALP assays revealed that OLFM4 overexpression rescued differentiation in FUT2‐knockdown HT29 cells, while OLFM4 knockdown attenuated differentiation in FUT2‐overexpressing SW480 cells (Figure 6D).

Scratch assay (Figure 6E) and Transwell invasion (Figure 6F) assay showed that OLFM4 knockdown in FUT2‐overexpressing SW480 cells significantly increased invasion and accelerated wound healing compared to FUT2 overexpression alone. Conversely, OLFM4 overexpression in FUT2‐knockdown HT29 cells decreased invasion and slowed wound healing versus FUT2 knockdown alone, indicating OLFM4 upregulation counteracts the promigratory and proinvasive effect of FUT2 deficiency.

Furthermore, analysis of EMT markers demonstrated that OLFM4 overexpression suppressed EMT in FUT2‐knockdown HT29 cells, while OLFM4 knockdown promoted EMT in FUT2‐overexpressing SW480 cells (Figure 6G,H). Similarly, stemness marker analysis indicated OLFM4 overexpression inhibited stemness in FUT2‐knockdown cells, and OLFM4 knockdown enhanced stemness in FUT2‐overexpressing cells (Figure 6I).

These results confirm OLFM4 as a key downstream target mediating FUT2’s regulation of colon cancer cell differentiation.

## 4. Discussion

Despite therapeutic advances, CRC remains highly aggressive, characterized by early metastasis and recurrence. These features correlate with poor tumor differentiation, which is associated with heightened stemness and EMT. Consequently, regulators of cancer cell differentiation represent promising therapeutic targets.

FUT2 mediates α‐1,2‐fucosylation in the intestinal tract, and it has been demonstrated that the fucosylation level of the colon was downregulated compared to the normal colon [3]. While implicated in lung cancer [31], squamous cell carcinoma [32], and CRC [22, 34], the mechanisms underlying FUT2’s role in CRC progression remain unclear. Notably, FUT2 deficiency reduces melanoma cell adhesion molecule (MCAM) fucosylation in intestinal epithelia, promoting CRC development [22]. Therefore, we can presume that FUT2 may regulate cancer cell differentiation through modulating the fucosylation level of certain molecules.

OLFM4, usually expressed in basal crypt cells, regulates intestinal stemness [35, 36] and tumor progression by modulating apoptosis, cell proliferation, adhesion, and metastasis [37]. Reduced OLFM4 correlates with colon cancer malignancy, poor prognosis, and enhanced cell migration [38], while patients with OLFM4‐positive colon cancer tend to have a better survival rate. Crucially, our study identifies FUT2 as an upstream regulator of OLFM4 fucosylation.

To confirm OLFM4 mediates FUT2’s effects, we rescued OLFM4 expression in FUT2‐manipulated cells. OLFM4 overexpression in FUT2‐deficient cells reversed EMT activation and suppressed invasion/migration, whereas OLFM4 knockdown in FUT2‐overexpressing cells promoted these processes. This demonstrates that OLFM4 is essential for FUT2‐mediated differentiation promotion, achieved through FUT2‐dependent OLFM4 fucosylation.

In conclusion, in this study, we demonstrated that FUT2 promoted colon cancer cell differentiation, and this effect was realized through the fucosylation level of OLFM4. The fucosylation level of OLFM4, regulated by FUT2, is positively correlated with cancer cell differentiation extent. Overall, fucosylation levels of different molecules are important in the prediction, monitoring, and treatment of colon cancer, and the results of this study may provide new insights for the treatment of colon cancer.

Limitations of this study include technical challenges in *in vivo* OLFM4 intervention, warranting future model refinement. Additionally, OLFM4 rescue incompletely restored invasion/migration to baseline, suggesting that FUT2 may regulate these processes through OLFM4‐independent mechanisms, necessitating further investigation.

**Nomenclature**
ALP: Alkaline phosphatase
AOM: Azoxymethane
β‐FGF: β‐fibroblast growth factor
DMEM: Dulbecco’s modified Eagle medium
DSS: Dextran sodium sulfate
EGF: Epidermal growth factor
PNGase F: N‐glycosidase F
EMT: Epithelial‐to‐mesenchymal transition
FBS: Fetal bovine serum
FUT2: Fucosyltransferase 2
GAPDH: Glyceraldehyde‐3‐phosphate dehydrogenase
IGF: Insulin‐like growth factor
MCAM: Melanoma cell adhesion molecule
OLFM4: Olfactomedin‐4
PBS: Phosphate‐buffered saline
qRT‐PCR: RNA extraction and real‐time quantitative PCR
UEA‐I: Ulex europaeus agglutinin‐I
2FF: 2‐fluorofucose

## Author Contributions

Caihan Duan: writing–original draft, data curation, methodology, investigation, formal analysis, and funding acquisition; Keyi Zhang: writing–original draft, data curation, and investigation; Lingzhi Hou: writing–original draft and investigation; Jiawei Chen and Jun Liu: review and editing and supervision; Huiying Shi and Chaoqun Han: writing–review and editing, supervision, resources, investigation, methodology, and funding acquisition. Caihan Duan and Keyi Zhang should be considered as joint first authors.

## Funding

This study was supported by the National Natural Science Foundation of China (Nos. 82270698, 82170570, 82470679, 82500681), the National Natural Science Foundation of Hubei Province (Nos. 2024AFB661 and 2025AFB034), and the China Postdoctoral Science Foundation (No. 2024M761069).

## Conflicts of Interest

The authors declare no conflicts of interest.

## Data Availability

The data that support the findings of this study are available from the corresponding author upon reasonable request.
